# MstX and a Putative Potassium Channel Facilitate Biofilm Formation in *Bacillus subtilis*

**DOI:** 10.1371/journal.pone.0060993

**Published:** 2013-05-30

**Authors:** Matthew E. Lundberg, Eric C. Becker, Senyon Choe

**Affiliations:** 1 Structural Biology Laboratory, The Salk Institute, La Jolla, California, United States of America; 2 Division of Biology, University of California San Diego, La Jolla, California, United States of America; Loyola University Medical Center, United States of America

## Abstract

Biofilms constitute the predominant form of microbial life and a potent reservoir for innate antibiotic resistance in systemic infections. In the spore-forming bacterium *Bacillus subtilis*, the transition from a planktonic to sessile state is mediated by mutually exclusive regulatory pathways controlling the expression of genes required for flagellum or biofilm formation. Here, we identify *mstX* and *yugO* as novel regulators of biofilm formation in *B. subtilis*. We show that expression of *mstX* and the downstream putative K+ efflux channel, *yugO*, is necessary for biofilm development in *B. subtilis*, and that overexpression of *mstX* induces biofilm assembly. Transcription of the *mstX-yugO* operon is under the negative regulation of SinR, a transcription factor that governs the switch between planktonic and sessile states. Furthermore, *mstX* regulates the activity of Spo0A through a positive autoregulatory loop involving KinC, a histidine kinase that is activated by potassium leakage. The addition of potassium abrogated *mstX*-mediated biofilm formation. Our findings expand the role of Spo0A and potassium homeostasis in the regulation of bacterial development.

## Introduction

Nearly all bacteria are capable of forming multicellular communities through complex signaling events that lead to differentiation into a myriad of cell types. These sessile, surface-attached bacterial populations, referred to as biofilms, create an elaborate extracellular matrix comprised of protein and exopolysaccharides that enhance survival in a nutrient-depleted state and mediate attachment to surfaces [1], [2]. Cells lacking either component of the extracellular matrix form flat, featureless colonies devoid of complex architecture and they fail to adhere to surfaces [3]. A signature feature of biofilm communities is their increased resistance to antibiotics and environmental stresses; both features make them particularly problematic in clinical and industrial settings. For instance, biofilms constitute over 65% of bacterial infections and represent a formidable source of innate multidrug resistance [4]. A small percentage of bacteria in a biofilm give rise to dormant, persistent cells that are recalcitrant to antibiotic treatment. The molecular mechanisms by which biofilms acquire antibiotic resistance have only recently begun to emerge [5]–[7].

The Gram-positive bacterium *B. subtilis* is capable of forming thin, floating biofilms at the liquid-air interface (pellicles) with sporulation at apical tips that project into the air [8]. Previous studies have identified multiple transcriptional regulatory networks that give rise to multicellular communities in *B. subtilis*. These pathways include the regulatory proteins Spo0A, σ^H^, the transition state regulator AbrB, the master regulator for biofilm formation SinR, and Slr [8]–[12]. Biofilm assembly requires expression of the 15-gene operon *epsA-O,* which encodes enzymes that synthesize the exopolysaccharide layer, the *tapA*-*sipW*-*tasA* operon, which encodes the protein component, and BslA, a protein that forms a hydrophobic layer on the surface of biofilms. SinR is a transcription factor that directly represses exopolysaccharide production and the flagellar motor inhibitor EpsE during exponential growth [13]. It also inhibits Slr, a transcriptional factor that activates biofilm genes while repressing motility [14]. The balance between SinR and Slr activity depends on Spo0A-P accumulation, which allows production of SinI, an inhibitor of SinR, which therefore turns on matrix production and turns off motility [12]. The switch between motility and biofilm formation therefore critically depends on the phosphorylation state of Spo0A, which is controlled by a variety of kinases and phosphatases that respond to different stimuli including oxidative stress, K+ leakage, osmotic pressure, and malic acid ([15]–[17]. These kinases (KinA, KinB, KinC, and KinD) help facilitate biofilm formation through spatial regulation but can be partially redundant through signaling overlap [18].

**Figure 1:**
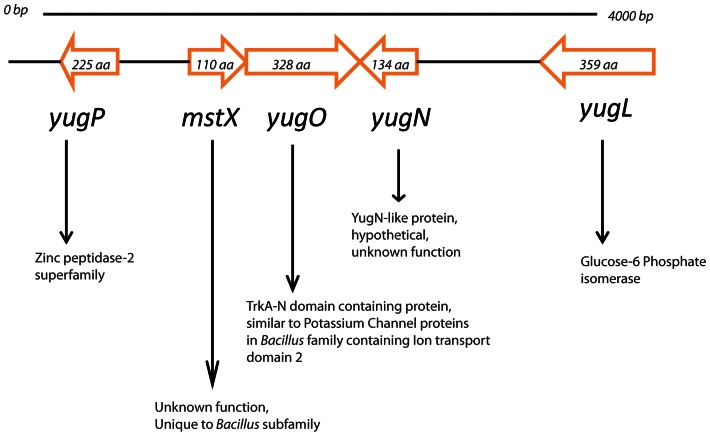
Diagram of the genetic organization of the chromosomal regions surrounding *mstX* and *yugO.* Sequence homology was identified by BLAST.

Mistic (MstX) is a unique protein found in a small number of *Bacillus* species, including *B. subtilis*, that enables high-level, heterologous expression and targeting of integral membrane protein sequences to cell membranes when fused to the N-terminus of a cargo protein construct [19]. In spite of its small and highly acidic nature, MstX associates with the membrane, presumably through autonomous association with the phospholipid bilayer, thereby bypassing or facilitating the traditional secretory apparatus [19]. The MstX homologues in *Bacillus atrophaeus*, *Bacillus mojavensis*, and *Bacillus licheniformis,* like *B. subtilis,* facilitate heterologous integral membrane protein expression when used as part of a fusion construct [20]. Furthermore, in all cases, *mstX* homologues precede a putative potassium ion channel *yugO,* suggesting that the MstX protein might be involved in membrane insertion of YugO (Figure 1). No similar sequence with a known function exists, raising the question as to what function MstX might serve in *Bacillus subtilis*.

The initial goal of the present work was to elucidate the function of *mstX* in the Gram-positive bacterium, *Bacillus subtilis*. During the course of this investigation, we discovered novel roles for *mstX* during biofilm development. We show that *mstX* is necessary for robust biofilm formation. The *mstX* promoter is regulated by SinR, the master regulator for biofilm formation, and induces biofilm formation at least partially through KinC mediated phosphorylation of Spo0A, and correspondent increases in expression of the regulators *abbA* and *sinI*. A mutation in SinR proved epistatic to the *mstX* biofilm film defect, restoring both colony morphology and pellicle formation in a double mutant. Supplementation of media with potassium or disruption of the downstream putative potassium ion channel abrogated *mstX*-mediated biofilm formation, illustrating the importance of KinC activation and potassium in biofilm development. These data suggest that *mstX* operates through a potassium efflux-driven positive feedback loop that enhances biofilm formation in *B. subtilis*.

## Methods and Materials

### Strains, media, and culture conditions

The parent strains for all experiments were either *B. subtilis* strains PY79 or NCIB3610 wild strain [8], [21]. Deletion mutants were created by long-flanking homology PCR or by standard cloning procedures [22]. A *loxP-kan-loxP* cassette was used to construct the *mstX* deletion, and after integration into the *B. subtilis* chromosome, the cassette was removed by Cre-mediated excision [23]. The IPTG-inducible expression strain, *P_spac_*-*mstX*, was constructed by cloning a full-length copy of the *mstX* coding region downstream of the *P_spac_* promoter and subsequent integration at the *lacA* locus in a *mstX::loxP* mutant. *P_spac_-mstX M75A* was obtained through site-directed mutagenesis of the resulting plasmid as described and introduced into a *mstX::loxP* mutant [24]. Additional details pertaining to strain construction can be found in the Supplemental Methods and Materials section (Text S1, Table S1).

### Biofilm growth and crystal violet assay

Biofilm growth and crystal violet assays for PY79 strains were performed essentially as described by Hamon and Lazazzera [9]. *Bacillus subtilis* starter cultures were grown to OD_600_ 0.3 at 37°C and added to polyvinylchloride microtitre plates (Fisher scientific) at a final OD_600_ of 0.01. Biofilm growth media was Luria-Bertani medium in addition to 0.15 ammonium sulfate, 100 mM potassium phosphate pH 7.0, 30 mM sodium citrate, 1 mM MgSO_4_ and 0.1% glucose or MsGG [8]. Samples of 100 µl diluted cells were added to 96-well PVC microtitre plates and incubated under stationary conditions at 30°C. 24 h after inoculation, we mixed the cultures by pipetting up and down as a means of oxygenating the cells. In addition, spent growth medium was exchanged for fresh biofilm growth medium. 72 h after inoculation and growth at 30°C, liquid medium was removed and wells were washed with fresh biofilm growth medium. Cells that had adhered to the wells were stained with 0.1% crystal violet at room temperature for 20 min. Excess crystal violet was then removed and adherent cells were washed with biofilm growth medium. The crystal violet that had stained the cells was solubilized in 200 ml of 80% ethanol and 20% acetone. Biofilm formation for each well was quantified by measurement of OD_570_ using a spectrophotometer. For characterizing biofilm growth in the NCIB3610 strain, cells were grown to OD_600_ 0.8 in LB and spotted with 2 µl on MsGG plates or inoculated in MsGG-containing wells [8]. Plates were grown for three days at 22°C and pellicles were then photographed with a Nikon Coolpix S4300 camera under special lighting. Wells measured approximately 3 cm in diameter.

### Chromatin Immunoprecipitation (ChIP)

*B. subtilis* strains were grown in Luria-Bertani medium to OD_600_ 0.5. Cultures were incubated for 30 min at room temperature. All chromatin immunoprecipitations were performed essentially as described [25]. Formaldehyde at final concentration of 1% and NaPO_4_ at final concentration 10 mm were added to 50 ml of cells grown in LB and 0.1 mM IPTG at OD_600_ 0.5. A *B. subtilis* PY79 background strain (MEL102) containing a *sinR-FLAG* gene fusion was created by cloning the complete sequence of *SinR-FLAG* into pMUTIN4 and by single-crossover recombination. The cross-linking reaction was terminated by the addition of glycine to a final concentration of 200 mM. Following cross-linking, cells were collected by centrifugation and were washed with TBS 50 mM Tris-HCl pH 7.5, 150 mM NaCl). The cells were then suspended in 1 ml ice cold lysis buffer [50 mM Tris-HCl with 1 mM EDTA, 5 mg/ml lysozyme and protease inhibitor cocktail (Roche)] and incubated for 30 min at 37°C. After lysis, Triton X-100 at final concentration 1% and sodium deoxycholate at final concentration 0.1% were added. The DNA was sheered by sonication to 500–1000 base-pairs, as determined by agarose gel electrophoresis. Insoluble cell debris was removed by centrifugation and the 50 µl supernatant was removed and added to 200 µl TES buffer (50 mM Tris-HCl pH 7.5, 10 mM EDTA, 1% SDS). DNA extracted from the insoluble cell debris was used as the “total DNA” control.

Protein and protein-DNA complexes were incubated (4°C O/N) with 5 µg monoclonal rabbit anti-FLAG antibody (Sigma-Aldrich) followed by incubation for 1 h at 4°C with Protein A-Sepharose beads (Sigma-Aldrich). Complexes were collected by centrifugation and washed 4 times (5 min at room temperature) with 1.5 ml Wash Buffer A (5 mM Tris-HCl pH 7.5, 500 mM NaCl, 1 mM EDTA, 1% Triton X-100, Roche protease inhibitors), twice (5 min at room temperature) with 1.5 ml of Wash Buffer B (50 mM Tris-HCl pH 8.0, 1 mM EDTA, 500 mM LiCl, 0.5% NP-40, 0.5% sodium deoxycholate) and once (5 min at room temperature) with TE Buffer (10 mM Tris-HCl pH 7.5, 1 mM EDTA). Protein and protein-DNA complexes were eluted from the beads by the addition of 100 µl of TES Buffer (50 mM Tris-HCl pH 7.5, 10 mM EDTA, 1% SDS) and incubation at 65°C for 15 min. The beads were removed by centrifugation and the eluate was transferred to a fresh tube and re-extracted with 150 µl of TES. The eluates as well as the “total DNA” sample were incubated overnight at 65°C. The DNA was subsequently extracted with phenol chloroform extraction and ethanol precipitation, with the resulting DNA resuspended in 100 µl TE buffer.

PCR with the listed primers (see Table S2) were carried out w/*Taq* DNA polymerase using standard PCR reaction conditions, with 1 µl of the DNA from the precipitation used as template. 1/100 µl of the “total DNA” was used for comparison. 25 amplification cycles were performed and the resulting PCR products were analyzed by agarose gel electrophoresis and ethidium bromide staining.

### RT-PCR and quantitative RT-PCR analysis

We used standard methods for analyzing gene expression via RT-PCR and quantitative RT-PCR, with some modifications [26]. For qRT-PCR analysis of *sinI*, *abbA*, *mstX*, *yqxM* and *sigA*, we used the primers listed in Table S2. The constitutively expressed *veg* gene (also known as BSU00440) was used as a positive control for quantifyinggene expression, as described by Hamon and colleagues [10]. For RT-PCR analysis of early log, stationary growth and biofilm growth cultures, we collected cells grown in LB medium at OD_600_ 0.3–0.5, OD_600_ 1.0, and microtitre plates. Cells grown as a biofilm were collected and characterized according to the growth protocol described above. RNA were isolated using RNAeasy miniprep columns (Qiagen) with the resulting RNA subjected to DNAse I digestion for 1 hr in order to remove contaminating chromosomal DNA. At this point, a “no reverse transcriptase” negative control was aliquoted, diluted 1/1000, and stored at −80°C. Following DNAse I digestion, cDNA were synthesized with Superscript II reverse transcriptase and a random hexamer primer according to standard protocol (Invitrogen). Following reverse transcription, the mixture cDNA and RNA were digested with RNAse to remove contaminating RNA. The solution was diluted by 1/1000 in Tris-HCl pH 8.5 buffer and stored at −80°C prior to use.

For RT-PCR, we added 1 µl cDNA or 1 µl from the no RT negative control to 100 µl GoTaq green master mix (Promega), applied 23 cycles, and analyzed by agarose gel electrophoresis. For real-time PCR, *abbA, sinI, kinC,* and *yqxM* transcription levels were standardized using *sigA* transcription and the ^ΔΔ^Ct method for quantification [27], [28]. 1 µl of cDNA preparation were added to 25 µl SYBRgreen mastermix (Invitrogen) and cycled 40 times in an ABI7900HT thermocycler. Data were averaged across three independent trials. Primer sequences are shown in Table S2.

## Results

### MstX and YugO promote biofilm formation in domesticated and undomesticated *B. subtilis* strains

**Figure 2:**
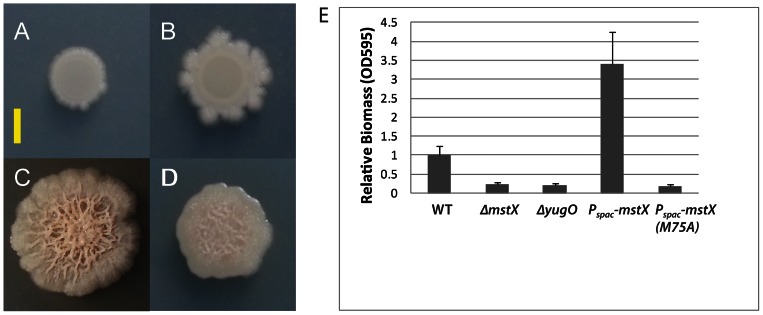
Alterations in colony morphology and biofilm formation related to *mstX* expression in the domesticated strain PY79. Images show colony morphology after 1 days of growth on MsGG medium at 30°C. Scale bar corresponds to approximately 3 mm. (A) Colony morphology of *B. subtilis* PY79. (B) Colony morphology of *B. subtilis* PY79 *ΔmstX* (MEL64). (C) Colony morphology of *B. subtilis* PY79 domesticated strain after IPTG induction of *mstX* (*lacA::P_spac_-mstX-erm;* MEL66). (D) Colony morphology of *Bacillus subtilis* PY79 after IPTG induction of *mstX* (*M75A)* (*lacA::P_spac_-mstX (M75A)-erm*; MEL67). (E) Microtitre crystal violet staining assay for WT, *ΔmstX*, *lacA::P_spac_ –mstX-erm*, and *lacA::P_spac_ -mstX (M75A)-erm* strains (strains PY79, MEL64, MEL66 and MEL67). Error bars represent standard error calculated from three independent experiments.

In order to elucidate the function of *mstX* in *Bacillus subtilis*, we first examined the effects of *mstX* overexpression and *mstX* deletion (*ΔmstX*) on growth. To do so, we constructed a strain expressing *mstX* under an IPTG-inducible promoter (*P_spac_*), integrated this construct into the chromosome of the domesticated strain PY79 with a *ΔmstX::loxP* mutation, and plated the strain in the presence and absence of 200 µM IPTG, on LB agar plates (1.5%). Induction of *mstX* did not impair growth (Figure S1) but gave rise to architecturally complex colonies (Figure 2C), whereas the corresponding wild-type and *ΔmstX* mutant strains formed colonies devoid of the thick exopolysaccharide layer that typifies biofilm formation (Figure 2A and Figure 2B). The *mstX* colonies showed a slight increase in motility but were otherwise indistinguishable from the wild-type strain (Figure 2B). These observations indicate that expression of *mstX* promotes the formation of architecturally complex colonies in a domesticated strain (PY79) that is otherwise incapable of forming these colonies or in forming robust biofilms.

To ensure that the observed enhancement of colony architecture was a product of *mstX* functionality as opposed to an unintended consequence of protein overexpression, we created a strain bearing an alanine substitution at a methionine residue (M75A) that has previously been shown to be essential for the ability of MstX to support high levels of membrane protein expression [19]. When expressed and purified heterologously in *E. coli*, MstX (M75A) mutant variants form more soluble oligomers that fail to associate with the membrane or enhance expression of cargo proteins [29]. In agreement with our *E. coli* expression results, the resulting *P_spac_-mstX* (*M75A)-erm* expression strain in a *ΔmstX::loxP* mutant background failed to form rough colonies in the presence of IPTG, in stark contrast to the *P_spac_-mstX* strain (Figure 2D). However, the *P_spac_-mstX* (*M75A) B. subtilis* mutant did not restore the wild-type colony morphology, as some roughness persisted. It is probable that the *mstX* (M75A) strain remained at least partially functional relative to the deletion strain. Functional expression of the *mstX* gene appears to play a significant role in producing the complicated colony architecture observed when it is overexpressed in the domesticated strain PY79.

We postulated that *mstX* might also be necessary for biofilm formation, a process that is associated with the ability to form architecturally complex colonies. We therefore quantified biofilm formation in a domesticated strain via a polyvinyl-chloride crystal violet assay [9], [30]. The assay measures the ability of bacterial cells to adhere and retain to a surface after washing, thereby approximating overall biofilm mass. The *ΔmstX* mutant and a *P_spac_*-*mstX(M75A)* mutant decreased biofilm formation over three-fold relative to the wild-type strain (Figure 2E). In the *P_spac_-mstX* strain, IPTG induction significantly enhanced biofilm formation in excess of the wild-type. These results show that *mstX* is both important for biofilm formation and that it appears to stimulate biofilm formation when expressed at high levels. MstX is a unique and relatively small protein with few orthologues in other species [20], and it is improbable that a protein with minimal genetic conservation is responsible for biofilm formation. However, *mstX* is immediately upstream of *yugO* in a two gene operon. YugO is a putative potassium efflux channel that contains a highly conserved sequence motif shared among many other prokaryotic potassium channels. Due to its location adjacent to *mstX* and the previously described role for potassium efflux in biofilm assembly [15], we questioned if *yugO* is also involved in biofilm formation.

**Figure 3:**
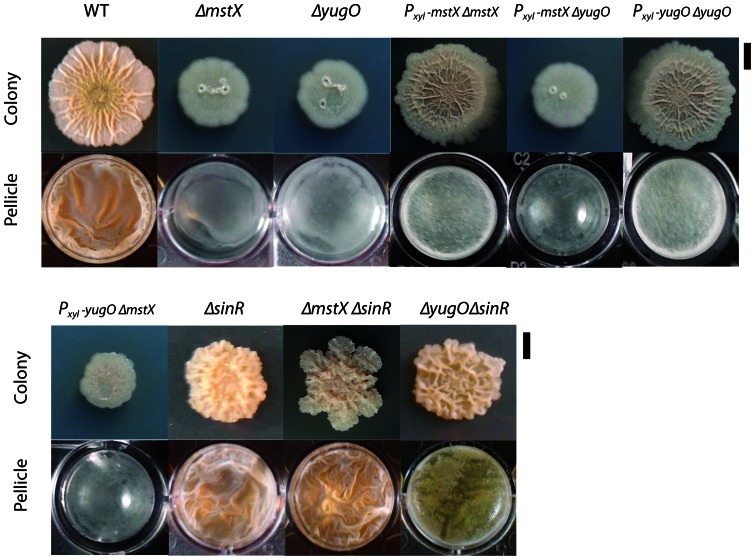
The *mstX* and *yugO* genes regulate biofilm formation in undomesticated *Bacillus subtilis* NCIB3610. Top rows of images show colony morphology after 3 days of growth on MsGG medium at 22°C; bottom rows show pellicle formation in MsGG medium after 72 hours of growth at 22°C. Strains used include NCIB3610 (wt), MEL240 (*ΔmstX::kan*), MEL239 (*ΔyugO::kan)*, MEL422 (*ΔmstX::kan, amyE::P_xyl_ –mstX-spc)*, MEL421 (*ΔyugO::kan, amyE::P_xyl_ –mstX-spc)*, MEL218 (*ΔsinR::spc*), MEL425 (*ΔmstX::kan*, *ΔsinR::spc*), MEL424 (*ΔyugO::kan*, *ΔsinR::spc*), MEL430 (NCIB3610 *P_xyl_-yugO-spc, Δyugo::kan*), and MEL431 (**NCIB3610** ***P_xyl_-yugO-spc, ΔmstX::kan***). The *ΔmstX* and *ΔyugO* mutations reduce colony architecture and pellicle formation, which is rescued by the *sinR* mutation. Microtitre wells measure approximately 3 cm in diameter.

These findings led us to hypothesize that *mstX* and *yugO* play a role in *B. subtilis* biofilm formation in an undomesticated strain that produces more robust biofilms than PY79. We transferred the aforementioned mutations and a *yugO* deletion (*ΔyugO*) into the undomesticated strain NCIB 3610 to see if we could replicate our results from the PY79 strain. Indeed, mutations in *mstX* and *yugO* decreased biofilm formation in both the colony architecture and pellicle formation assays (Figure 3). The resulting mutants formed thin and detached surface pellicles that failed to recapitulate the observed phenotype in the wild-type strain. The introduction of an extra copy of *mstX* or *yugO* at the *amyE* locus under the control of the xylose-inducible promoter (*P_xyl_*) rescued the *ΔmstX or ΔyugO* strain for its biofilm defect in the presence of 0.5% xylose. However, both *mstX* and *yugO* were required in order to restore biofilm formation to wild-type levels (Figure 3), so it is likely MstX acts in conjunction with YugO in order to promote biofilm formation. In agreement with this conclusion, a *P_xyl_-mstX* failed to rescue a *ΔyugO* mutation and behaved the same as a strain missing *mstX*. Similarly, a *P_xyl_-yugO* failed to rescue a *ΔmstX* mutation and behaved the same as a strain missing *yugO* (Figure 3). MstX has previously been shown to enhance integral membrane protein expression when fused to a diverse number of heterologous proteins, including those of eukaryotic origin. We suggest that MstX likely plays a similar role in promoting the membrane insertion or expression of YugO, a putative potassium channel downstream of the *mstX* open-reading frame.

### A *ΔsinR* mutation restores biofilm formation and gene expression in *ΔmstX* and *ΔyugO* mutants

SinR is a key regulatory protein that represses biofilm assembly during growth and under non-biofilm promoting conditions [31]. The failure in biofilm assembly in the *ΔmstX* and *ΔyugO* mutants could either be due to a structural defect in biofilm assembly or to continued SinR activity during biofilm promoting conditions. To test this hypothesis, we examined the effect of the *sinR* mutation on colony architecture and pellicle formation in the *mstX* and *yugO* mutant strains. In agreement with previous observations, the wild-type NCIB3610 strain and sinR mutant strain exhibited complex colony architecture or wrinkled pellicles, respectively. In contrast, smooth nonstructured colonies were detected for the *mstX* mutant and *yugO* mutants (Figure 3). Introduction of a *sinR* mutation into strains with a *mstX* or *yugO* mutation restored biofilm formation. These data suggest that the *mstX* and *yugO* mutants fail to initiate biofilm assembly because SinR remains active, thus significantly repressing the genes responsible for assembly.

**Figure 4:**
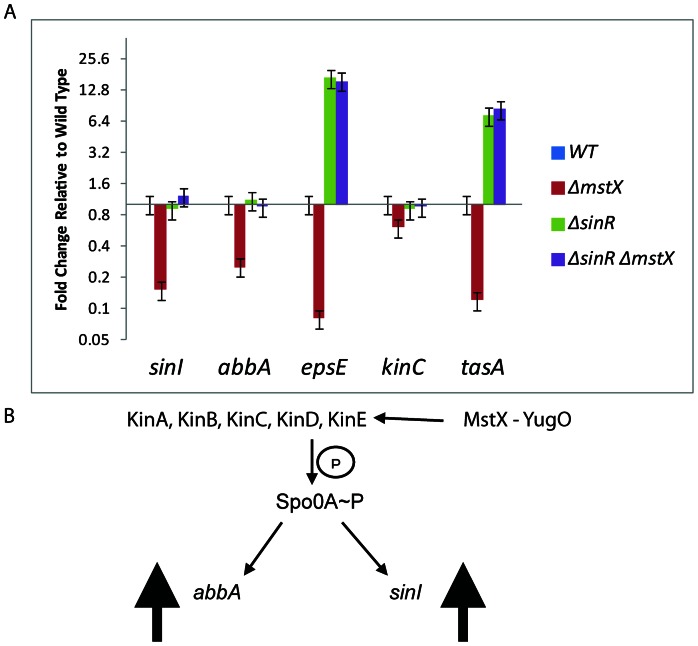
MstX negatively regulates parallel antirepressors involved in biofilm formation. (A) Quantitative RT-PCR analysis of *abbA, sinI, epsE, kinC,* and *tasA* in wild type, *ΔmstX*, *ΔsinR*, and *ΔsinR ΔmstX* double mutants (strains MEL65, MEL240, MEL423 and MEL 425) were grown in MsGG medium at 30°C and collected at 0.5–0.8 OD_600_. Error bars represent standard error calculated from three independent experiments. (B) Model for *mstX* activation of *kinC* with corresponding increases in *abbA* and *sinI* transcription.

Continued SinR activity in the *mstX* mutant should result in decreased expression of genes required for biofilm formation, such as *eps* and *tasA,* which are involved in matrix assembly, and this defect in gene expression should be rescued by the disruption of *sinR* repression. To test this hypothesis, we used qRT-PCR in the NCIB3610 background strain to examine the effects of MstX and SinR on expression of these genes and on the key regulatory genes *abbA*, *sinI* and *kinC*. We harvested total RNA from the designated strains grown in MsGG media (OD_600_ 0.5) in order to mimic biofilm forming conditions. The disruption of *mstX* expression significantly reduced the expression of the biofilm structural components, *epsE* and *tasA*, and the regulatory antirepressor genes, *abbA* and *sinI* (Figure 4, 5-fold). However, the upstream kinase *kinC* experienced only marginally decreased expression (less than 2-fold), indicating that most of the observed deficiency in biofilm formation is a byproduct of decreased antirepression of SinR and AbrB by lower levels of SinI and AbbA. Decreased antirepression of SinR would thus directly lead to decreased expression of downstream targets of SinR, such as *epsE* and *tasA*.

**Figure 5:**
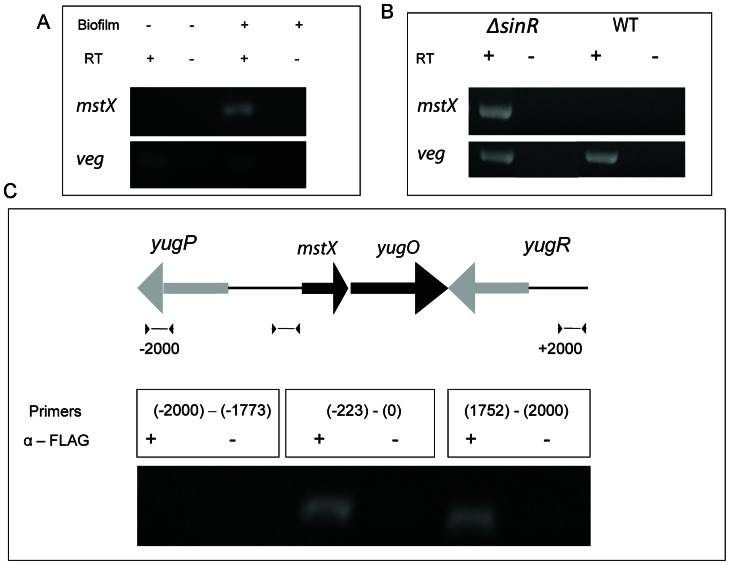
Temporal expression of *mstX* and regulation by the transcriptional repressor SinR. (A) Comparison of *mstX* expression during log growth and biofilm growth using RT-PCR analysis in strain MEL63. The *veg* gene (BSU00440) is a constitutively expressed gene frequently used as a positive control for RT-PCR. (B) Comparison of *mstX* gene expression in the presence and absence of the transcriptional repressor SinR using RT-PCR in strains MEL63 and MEL73. Cultures were collected during log growth. (C) Chromatin immunoprecipitation assay of strain MEL102 shows that SinR-FLAG binds near the *mstX* promoter during exponential growth.

The expression of *sinI* and *abbA* are controlled by the sporulation and biofilm regulatory factor, Spo0A. Spo0A is a bistable regulator that is activated heterogeneously across the cell population. Although SinR predominates over SinI even in media that promotes the derepression of the *eps* matrix operon, *sinI* is selectively overexpressed in a distinct subpopulation of cells that ultimately gives rise to a biofilm. Increased phosporylation of Spo0A in this subpopulation contributes to increased expression of SinI and consequent SinR repression. These results suggest that the *yugO* and *mxtX* mutants affect the levels of Spo0A-P, most probably through increased activity of one of the upstream kinases, KinA, KinB, KinC, or KinD (Figure 4B).

### The *mstX-yugO* operon is selectively expressed during biofilm assembly

If MstX and YugO are involved in biofilm assembly, then they should be produced during conditions that promote biofilm production. We therefore assessed temporal expression of *mstX* in *B. subtilis*. We used RT-PCR analysis to determine *mstX* transcript levels during logarithmic growth phase and stationary cultures grown as a biofilm on PVC plates. *B. subtilis* cultures grown in LB media or under biofilm-forming conditions were collected and analyzed by RT-PCR. Primers were selected for amplifying ∼200 bp regions of *mstX* and *veg*, a constituitively expressed gene that functioned as an internal control [10]. RT-PCR revealed low expression levels in early and mid-logarithmic growth. However, *mstX* transcription was significantly upregulated in RNA harvested from *B. subtilis* biofilms (Figure 5A). Thus, *mstX* is expressed during biofilm formation, consistent with the role it plays in promoting biofilm formation.

### SinR directly regulates the *mstX-yugO* promoter

Investigation of the *mstX*-*yugO* promoter region indicated that it possesses a putative SinR binding site (GTTCTTT) at −65 base pairs relative to the likely *mstX* translational start codon, suggesting that the operon might be regulated by SinR. We therefore tested if SinR represses *mstX* expression. We constructed a *ΔsinR* mutant strain and examined *mstX* expression in the absence of SinR *in vivo*. RNA was harvested at late stages of logarithmic growth for analysis (OD_600_ ∼0.8) from a PY79 wild-type strain and a *ΔsinR::neo* deletion strain (Materials and Methods). RT-PCR demonstrated that *mstX* was expressed during logarithmic growth phase in the *sinR* mutant but not in the wild-type strain (Figure 5B). In the wild-type strain, no *mstX* transcripts were detected during logarithmic growth. These results indicate that SinR represses *mstX* expression during growth and that the alleviation of SinR repression induces *mstX* expression during biofilm formation.

To determine if SinR directly binds the *mstX* promoter, we constructed a strain with a FLAG-tagged copy of SinR for chromosome immunoprecipitation experiments (ChIP). A copy of *sinR-FLAG* was cloned in pMUTIN4 and integrated into the chromosome through single recombination event and erythromycin selection. ChIP experiments were performed with samples harvested during logarithmic growth, when *mstX* is expressed at low levels. Samples were formaldehyde crosslinked and purified on a FLAG affinity column and the regions of interest amplified by PCR (∼200 bp target sequence). The PCR primers were designed to amplify the *mstX-yugO* promoter region, as well as two flanking genes, *yugP* and *yugR* (Figure 5C). PCR failed to detect an interaction between *yugP* and SinR, but did reveal an interaction near the *mstX*-*yugO* promoter region and within *yugR*. The inability of SinR-FLAG to bind *yugP* sequence demonstrates the specific binding of our protein construct. Thus, SinR appears to have multiple binding sites near the *mstX-yugO* region.

### Potassium and a *kinC* mutant abrogate biofilm formation in a MstX-YugO expression strain

**Figure 6:**
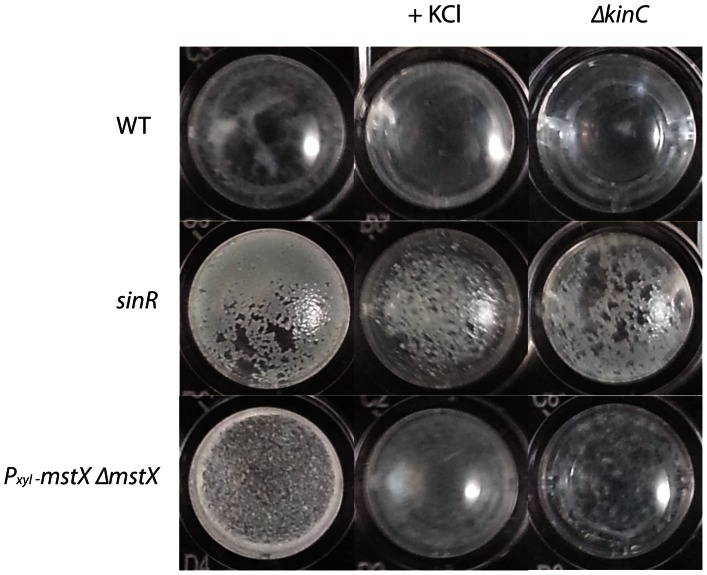
Addition of 150 mM Potassium chloride or the *kinC* deletion abrogates pellicle formation after *mstX* overexpression and in the *sinR* strain in LB medium that does not normally support biofilm formation in the NCIB3610 background strain. Strains used includes MEL65 (wt NCIB3610), MEL423 (*ΔsinR*) and MEL428 (*ΔsinR, ΔkinC),* MEL422 *(amyE::P_xyl_-mstX-spc)* and MEL429 (*amyE::P_xyl_-mstX-spc, ΔkinC::cm).* Xylose-inducible strains were grown in the presence of 0.5% xylose.

Potassium efflux has previously been shown to induce biofilm formation in *B. subtilis* by activating the Spo0A kinase KinC, suggesting that MstX and the YugO K+ efflux pump might induce biofilm formation by activating KinC. To test this hypothesis, we first examined if KinC is necessary for biofilm formation when *mstX* is overexpressed in LB plates. The wild-type NCIB3610 strain does not form robust biofilms when grown in LB media (Figure 6). However, when *mstX* is introduced, the strain can form a solid pellicle when induced. Deletion of *kinC* or addition of KCl disrupted the biofilm phenotype of a *mstX* overexpression strain grown in microtitre plates (Figure 6). Minimal pellicle was formed compared to the wild-type strain or the strain without the *kinC* mutation (Figure 6). This result indicates that KinC and KCl are importantfor *mstX*-mediated biofilm formation and that *mstX* principally promotes biofilm formation through this pathway.

After examining the influence of *kinC* and KCl on *mstX*-mediated biofilm formation, we then sought to determine their interactions with SinR under limiting biofilm growth conditions. The introduction of *sinR* nullified the impact of a *kinC* mutation or the addition of KCl, implying that these effectors of biofilm growth act at least in part through direct or indirect inhibition of SinR. SinR is the master regulator for biofilm formation and a dominant player in the transition from planktonic to sessile states in *B. subtilis* [11]. Thus, it is probable that *mstX* acts at least in part by derepressing SinR during biofilm formation.

**Figure 7:**
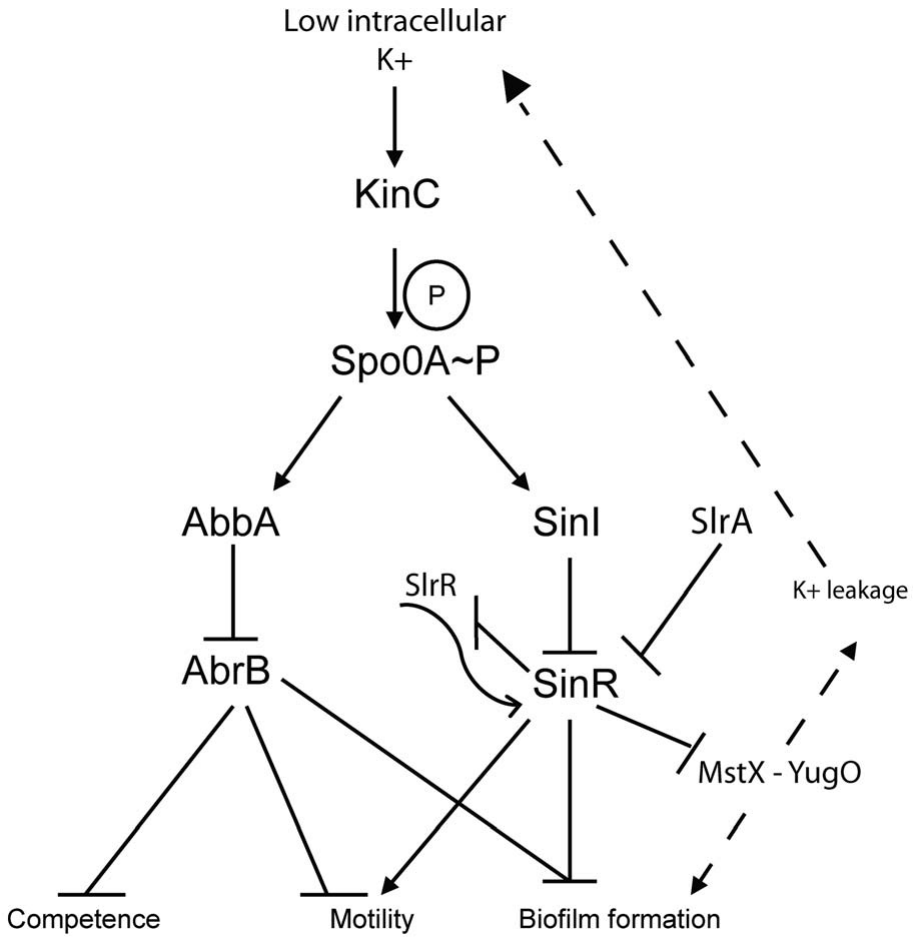
A positive autoregulatory loop involving MstX, YugO, potassium, and biofilm formation in *B. subtilis*. Our data suggest that MstX and YugO both positively regulate biofilm formation by inhibiting SinR (in a manner dependent on KinC and influenced by potassium) and that the expression of *mstX* and *yugO* are negatively regulated by SinR. This suggests that MstX and YugO participate in a positive feedback loop to lock a subpopulation of cells in the biofilm assembling state. We propose that MstX mediates the assembly of YugO, a putative potassium efflux channel, and that potassium leakage activates KinC [15], [35], [44].

The ability of certain small molecules to induce biofilm formation by stimulating K+ efflux is abrogated by high concentrations of extracellular potassium. Lopez and colleagues (2009) identified the kinase KinC as a critical component to potassium efflux biofilm assembly when subjected to small molecules that contribute to potassium leakage [15]. We proposed that biofilm induction by overexpression of *mstX* might also depend upon extracellular concentrations of potassium and that the addition of potassium would suppress the biofilm phenotype in an *mstX*-induced biofilm strain. To test this hypothesis, we grew an *mstX* overexpression strain in the presence of 150 mM KCl. In microtitre plates, both the wild-type and *mstX* overexpression strains failed to form robust pellicles in the presence of potassium (Figure 6). KCl at 150 mM KCl does not inhibit inhibit the growth of B. subtilis but does perturb biofilm formation (Figure S2). Thus, our results suggest that the effect of MstX and YugO on biofilm assembly is mediated by their ability to activate KinC, which in turn activates Spo0A to mediate the inhibition of SinR and the onset of biofilm gene expression. These findings further suggest that MstX promotes the assembly of active YugO. In this model, YugO would mediate K+ efflux, thereby activating KinC and leading to Spo0A-P production and biofilm assembly (Figure 7).

## Discussion

The data presented here indicate that the *mstX-yugO* operon, which was identified in a screen for putative potassium channels in *B. subtilis*, participate in biofilm formation. We previously noted that including *mstX* in overexpression plasmids greatly facilitated expression of the downstream integral membrane protein YugO [32] and that *mstX* family members displayed the ability to increase expression of multiple heterologous membrane proteins in *E. coli* when expressed as an N-terminal fusion protein to a cargo membrane protein [20]. MstX possesses no homology to proteins of known function, leaving open the question as to how MstX might function in *B. subtilis.* Our findings suggest that MstX and the putative potassium channel YugO positively regulate biofilm assembly in a pathway that depends on the key regulatory protein SinR.

The transcription factor SinR plays a critical role in regulating the transition from motile, logarithmic growth to sessile biofilms or pellicle structures in *B. subtilis* populations. SinR manages this switch in development by repressing the expression of genes required for the synthesis of the biofilm matrix. The activity of SinR is coordinated by antagonistic regulatory interactions between SinR and the antirepressors SinI or SlrR, with *slrR* also representing a target for repression by SinR (Figure 7). SlrR serves an additional function as a repressor of σ^D^-dependent genes for autolysis and motility, including the *hag* gene [33], [34]. Expression of SinR and its antagonist, SinI, is activated by the phosphorylated master regulator of sporulation, Spo0A. Spo0A is bistable and only phosphorylated in a fraction of the cell population, thereby influencing motility and biofilm formation by regulating the activity of SinR through an ON and OFF switch [33]. As conditions trigger an accumulation of phosphorylated Spo0A, an increasing number of cells repress SinR activity via antagonistic interactions with the antirepressors SinI and SlrR [35]. The consequent cellular inhibition of SinR enables the expression of genetic components that ultimately give rise to a biofilm.

Our findings implicate MstX and YugO in a positive autoregulatory cascade involving potassium, KinC activation, Spo0A phosphorylation and the alleviation of SinR mediated repression of biofilm formation in *B. subtilis*. Specifically, we demonstrate that *mstX* overexpression induces biofilm formation in a manner dependent on K+, YugO and KinC functionality. Furthermore, disruption of *mstX* or *yugO* abrogated biofilm formation in a manner that was suppressed by a *sinR* mutation, suggesting that MstX and YugO stimulate biofilm formation by inhibiting SinR, which represses biofilm formation. The *mstX* mutation also caused decreased expression of the *abbA* and *sinI* antirepressors. This suggests that in the absence of MstX, cells fail to accumulate sufficient SinI to overcome the SinR-mediated repression of biofilm formation.

This pathway is likely dependent upon potassium signaling either through MstX or, more likely, through a putative potassium ion channel encoded by the gene that lies downstream of *mstX*, *yugO* (Figure 1). The following lines of evidence support the hypothesis that MstX and YugO work together in potassium regulation: (i) disruption of *yugO* also generates a strain with similar biofilm phenotype as the *mstX* deletion strain, (ii) a *yugO* knockout, a *kinC* knockout, or elevated KCl concentrations ablate biofilm formation in an xylose-induced *P_xyl_-mstX* strain (Figure 6), (iii) *yugO* encodes a putative TrkA-domain containing potassium ion channel that was initially identified in a bioinformatic screen for potential potassium ion channels in *B. subtilis* [12] and it possesses sequence similarities to KefC, a widely distributed glutathione-S-conjugate gated K+ efflux system [36]–[39]. It is unclear whether MstX contributes to enhanced KinC activation through non-surfactant-mediated K+ ion leakage or via allosteric activation of the kinase through production of a small-molecule enhancer [40]. However, the minimal decrease in expression of *kinC* in a *mstX* deletion mutant and the phenotypic dependence on *yugO* point to a connection with its downstream gene, *yugO*. We therefore propose that MstX primarily operates by enhancing the functional expression of *yugO* (likely at the stage of membrane insertion [19]), facilitating potassium ion leakage through the putative YugO ion channel, activating KinC and biofilm formation.

Two previous reports have indicated the important role played by potassium in biofilm assembly in *B. subtilis*. First, Lopez and colleagues [15] identified potassium leakage and KinC activation as a critical regulator of biofilm formation in *B. subtilis.* In this pathway, Surfactin contributes to membrane permeabilization, potassium ion leakage and KinC activation responsible for biofilm formation. Deletion of the *srfA* genes required for surfactin production or the *kinC* gene that encodes the KinC kinase abrogated biofilm formation in *B. subtilis*. Second, Lopez and colleagues identified the potassium ion transport regulator KtrC as being instrumental in biofilm regulation included as part of a supplemental finding [15]. Deletion of *ktrC* gave rise to increased biofilm formation in a microtitre plate assay, as one would expect with elevated intracellular concentrations of K+ and decreased KinC phosphorylation of Spo0A (see Text S1, [15]).

Potassium ion channels are widely distributed among prokaryotic species and have been used as models for understanding potassium signaling in the excitation of nerves or muscles of multicellular organisms [30]. Core features of potassium channels, including gating principles and the structure of the selectivity filter, are highly conserved over evolutionary space. The structures and activities of potassium ion channels evolved long before the emergence of complex multicellular organisms and their use in neurophysiology. However, the roles of selective cation channels in microbial physiology remain largely unknown. Our finding ascribes a known physiological function to a potassium ion channel in a prokaryote. It is unlikely that *B. subtilis* is unique in utilizing potassium efflux for intracellular signaling. Potassium uptake and efflux systems have also been implicated as critical regulators for biofilm formation and pathogenesis in *Pseudomonas aeruginosa* [41]. In addition, the KefC system couples glutathione-adduct formation to cytoplasmic acidification by potassium transport, thereby protecting *Escherichia coli* from electrophilic attack [39]. The potential for ligand-gated ion channel transport to serve as a means for coordinating complex and spontaneous signal transduction throughout a cell without the necessity of protein synthesis provides a tantalizing explanation for the prevalence of cation channels in both prokaryotic and eukaryotic species.

The data presented in this study demonstrate that MstX and YugO influence the expression of key antirepressors involved in modulating the activity of the master regulator for biofilm formation, SinR and that the *mstX-yugO* operon is also regulatd by SinR. We find that *mstX-yugO* is selectively expressed during biofilm formation and that SinR directly represses *mstX* transcription during growth. We also show that KinC and YugO are required for facilitating biofilm assembly via MstX-mediated SinR derepression. Thus, the ability of MstX and YugO to induce biofilm formation via KinC and SinR together with the repression of MstX by SinR would provide the basis for positive autoregulation and enhancement of the regulatory cascade involved in biofilm formation (Figure 7).

Previously it has been shown that bistable gene expression can be influenced by any mutation that disrupts the regulators that control the transcription of the bistable target genes. One example is increased expression of the *sigD* gene results in the accumulation of active σ^D^. This is likely a product of an inadequate amount of the anti-sigma factor FlgM to inactivate the σ^D^ due to the shift in stoichiometry. As a result, expression of the σ^D^ regulon no longer exhibits dispersed bistability but occurs in all cells of the population [28], [34]. Similarly, a mutation in the putative phosphodiesterase *ymdB* shifts the stoichiometry of c-di-GMP toward a motile state and impairs biofilm formation [28]. MstX expression might operate in a similar fashion by increasing the expression of the antagonist SinI, thus decreasing the activity of the bistable regulator SinR.

The application of MstX to membrane protein overexpression in *Escherichia coli* has enabled the production of many eukaryotic membrane proteins and other challenging bacterial proteins [19], [42], [43]. Nevertheless, a complete explanation describing the mechanism that enables MstX-tagged overexpression of otherwise toxic membrane proteins in *Escherichia coli* or MstX-mediated biofilm induction in *B. subtilis* remains elusive. Preliminary structural analysis indicates that the oligomeric state of MstX may be instrumental for its membrane protein chaperoning properties [29]. MstX homologues in *Bacillus mojavensis*, *Bacillus licheniformis*, and *Bacillus atrophaeus* present different solubility profiles than *Bacillus subtilis* yet still facilitate membrane protein overexpression in *E. coli* [20], [29], [32]. In all cases, MstX homologues precede a putative potassium ion channel (*yugO*). It is plausible that MstX facilitates expression of the putative ion channel in these strains, thereby triggering the events that lead to biofilm formation.

In summary, our results identify *mstX* as a novel determinant of biofilm formation and antibiotic resistance in *B. subtilis* that is positively autoregulated through a genetic loop involving potassium, KinC, Spo0A activation, and SinR-mediated derepression. *MstX* expression was necessary and capable of inducing biofilm formation under nutrient conditions which would normally inhibit biofilm formation, rescuing an atavistic response in a domesticated *B. subtilis* strain. The addition of potassium or deletion of *yugO*, a putative potassium ion channel, abrogated *mstX*-mediated biofilm formation, highlighting the importance of potassium homeostasis for initiation of regulatory networks involved in development. Our finding raises the prospect for a broader role for potassium ion channel signaling in microbial physiology.

## Supporting Information

Figure S1 Growth of *B. subtilis* mutants in (a) LB or (b) MSgg media. Growth of the following strains was monitored: *B. subtilis* wt (NCIB3610), the *mstX*-deletion strain MEL (*mstX*), the *yugO*-deletion strain MEL (*yugO*), and the *mstX* overexpression strain MEL (*P_xyl-_mstX*). The wild-type strain and deletion strains were also grown in 0.25% xylose. A volume of 5 ml of xylose-free MSgg or LB medium was inoculated with fresh colonies and incubated overnight at 37°C. Roller flasks containing 5 ml of xylose-containing MSGG or LB were inoculated with diluted aliquots of the overnight culture (start OD600 0.01) and incubated at 37°C. Growth was monitored by optical density measurements. Values represent the mean of three independent trials.
(TIF)

Figure S2 **Growth of** ***B. subtilis*** **NCIB3610 wild-type strain in LB or LB media supplemented with 150** **mM KCl.** A volume of 5 ml of LB medium was inoculated with fresh colonies and incubated overnight at 37°C. Roller flasks containing 5 ml of LB were inoculated with diluted aliquots of the overnight culture (start OD600 0.01) and incubated at 37°C. Growth was monitored by optical density measurements. Values represent the mean of three independent trials.
(TIF)

Table S1 **Strain List.**
(TIF)

Table S2 **Primers used in this study.**
(TIF)

Text S1 **Materials and methods.**
(DOCX)
