# Supplementary figures and images for: MstX and a Putative Potassium Channel Facilitate Biofilm Formation in Bacillus subtilis
Source: PLoS One. 2013 May 30;8(5):e60993. doi: 10.1371/journal.pone.0060993 (PMC3667857; doi:10.1371/journal.pone.0060993)

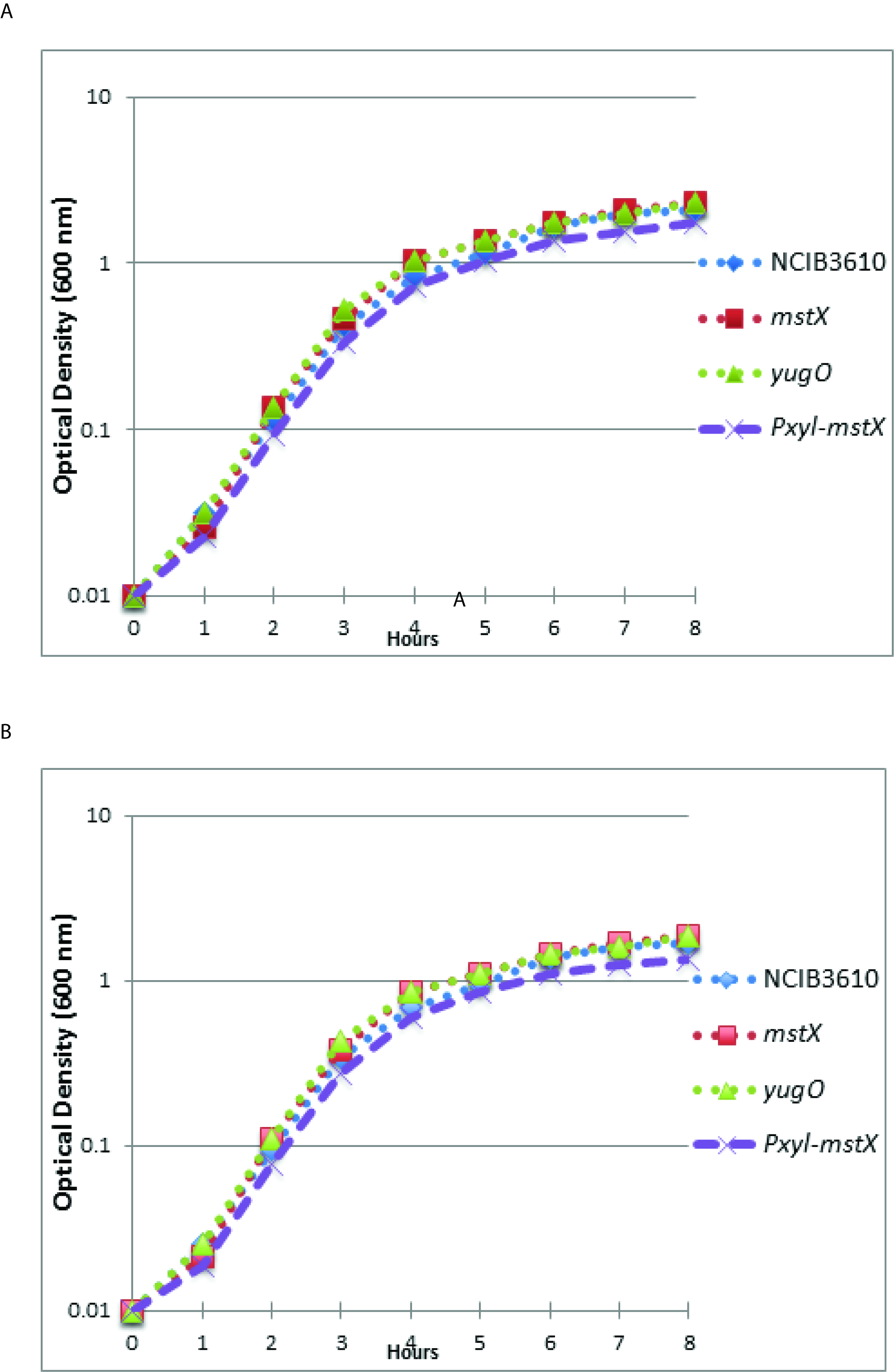

Supplement: Figure S1 — Growth of B. subtilis mutants in (a) LB or (b) MSgg media. Growth of the following strains was monitored: B. subtilis wt (NCIB3610), the mstX-deletion strain MEL (mstX), the yugO-deletion strain MEL (yugO), and the mstX overexpression strain MEL (Pxyl-mstX). The wild-type strain and deletion strains were also grown in 0.25% xylose. A volume of 5 ml of xylose-free MSgg or LB medium was inoculated with fresh colonies and incubated overnight at 37°C. Roller flasks containing 5 ml of xylose-containing MSGG or LB were inoculated with diluted aliquots of the overnight culture (start OD600 0.01) and incubated at 37°C. Growth was monitored by optical density measurements. Values represent the mean of three independent trials. (TIF) [file pone.0060993.s001.tif]

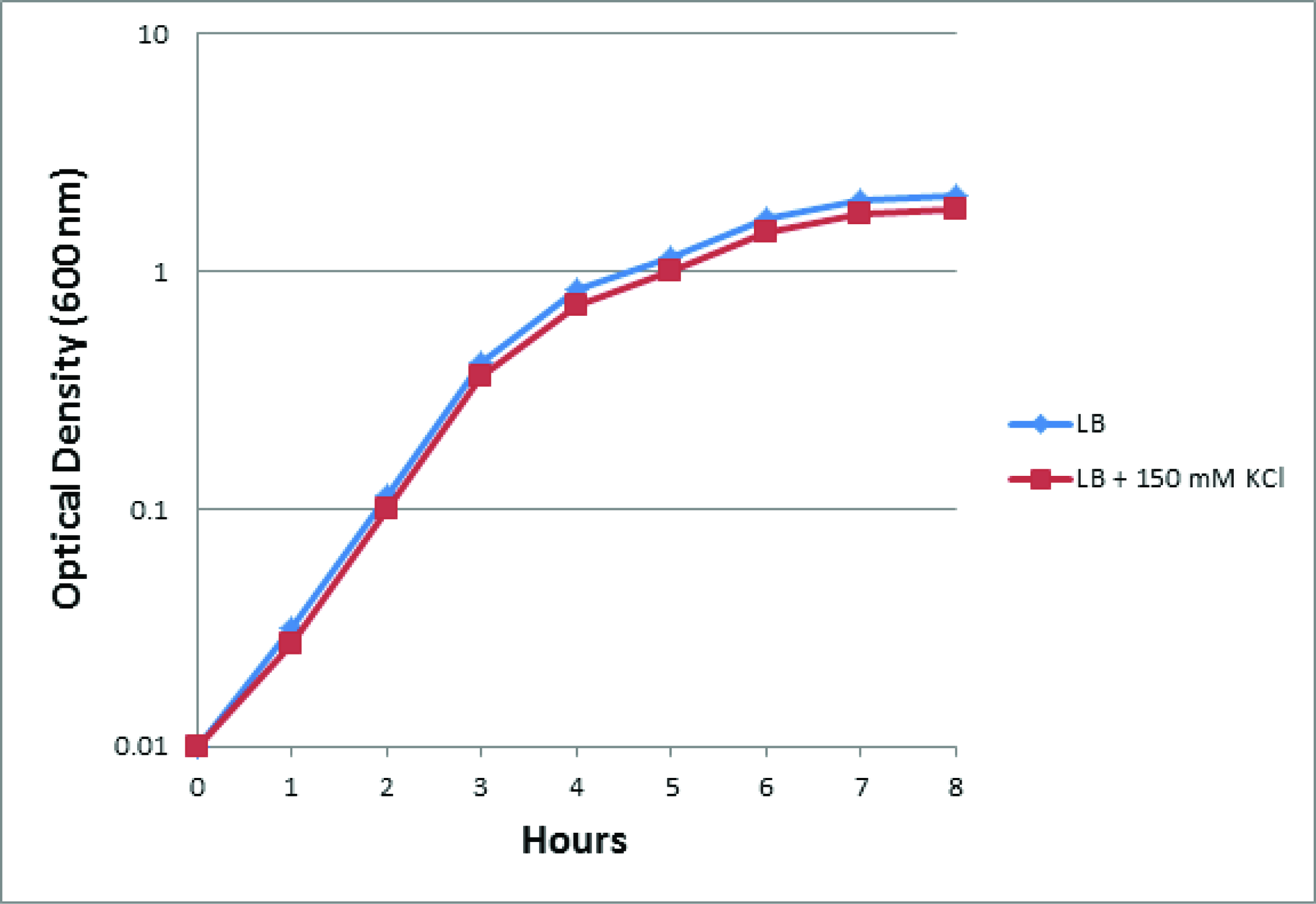

Supplement: Figure S2 — Growth of B. subtilis NCIB3610 wild-type strain in LB or LB media supplemented with 150 mM KCl. A volume of 5 ml of LB medium was inoculated with fresh colonies and incubated overnight at 37°C. Roller flasks containing 5 ml of LB were inoculated with diluted aliquots of the overnight culture (start OD600 0.01) and incubated at 37°C. Growth was monitored by optical density measurements. Values represent the mean of three independent trials. (TIF) [file pone.0060993.s002.tif]

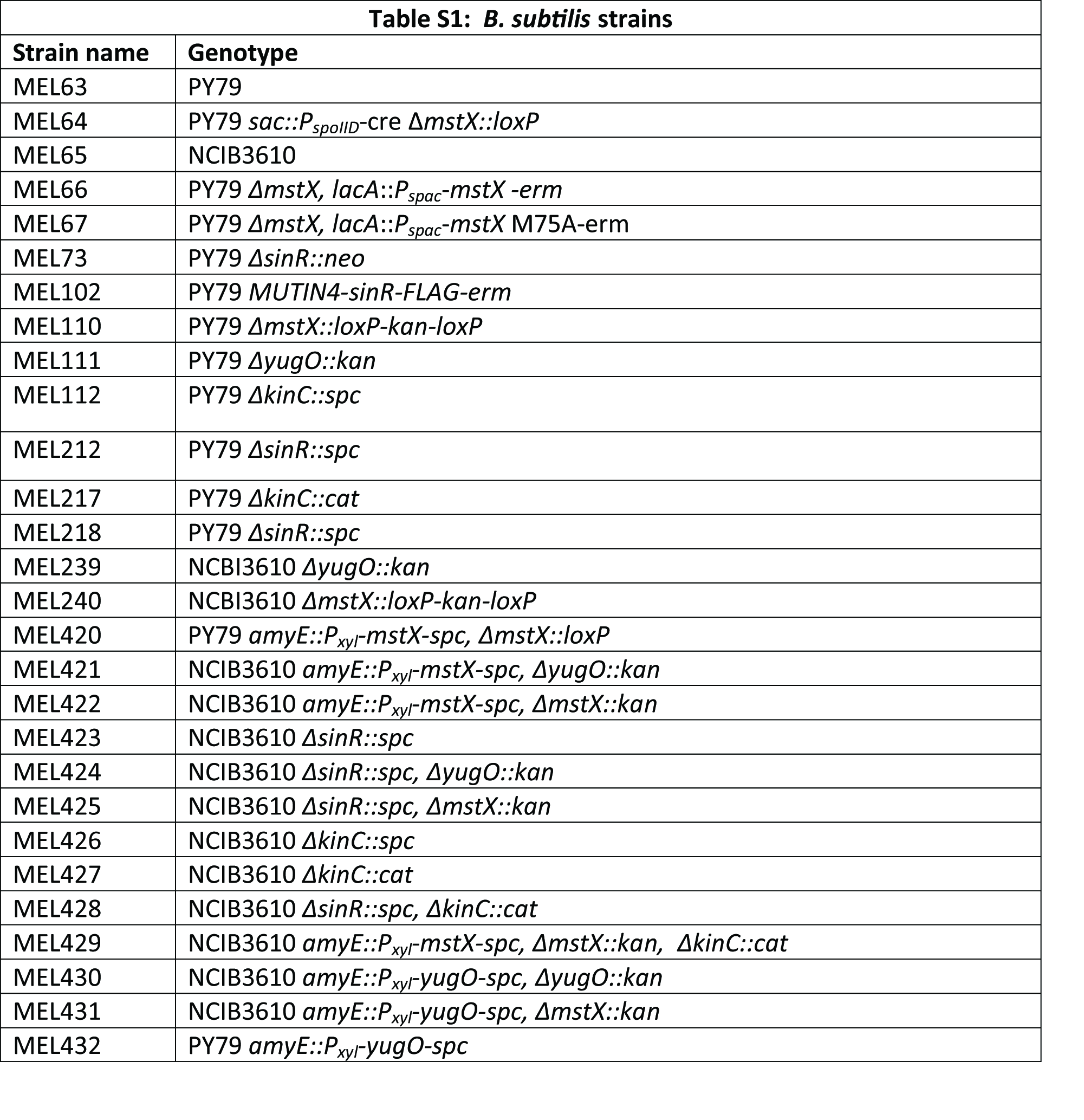

Supplement: Table S1 — Strain List. (TIF) [file pone.0060993.s003.tif]

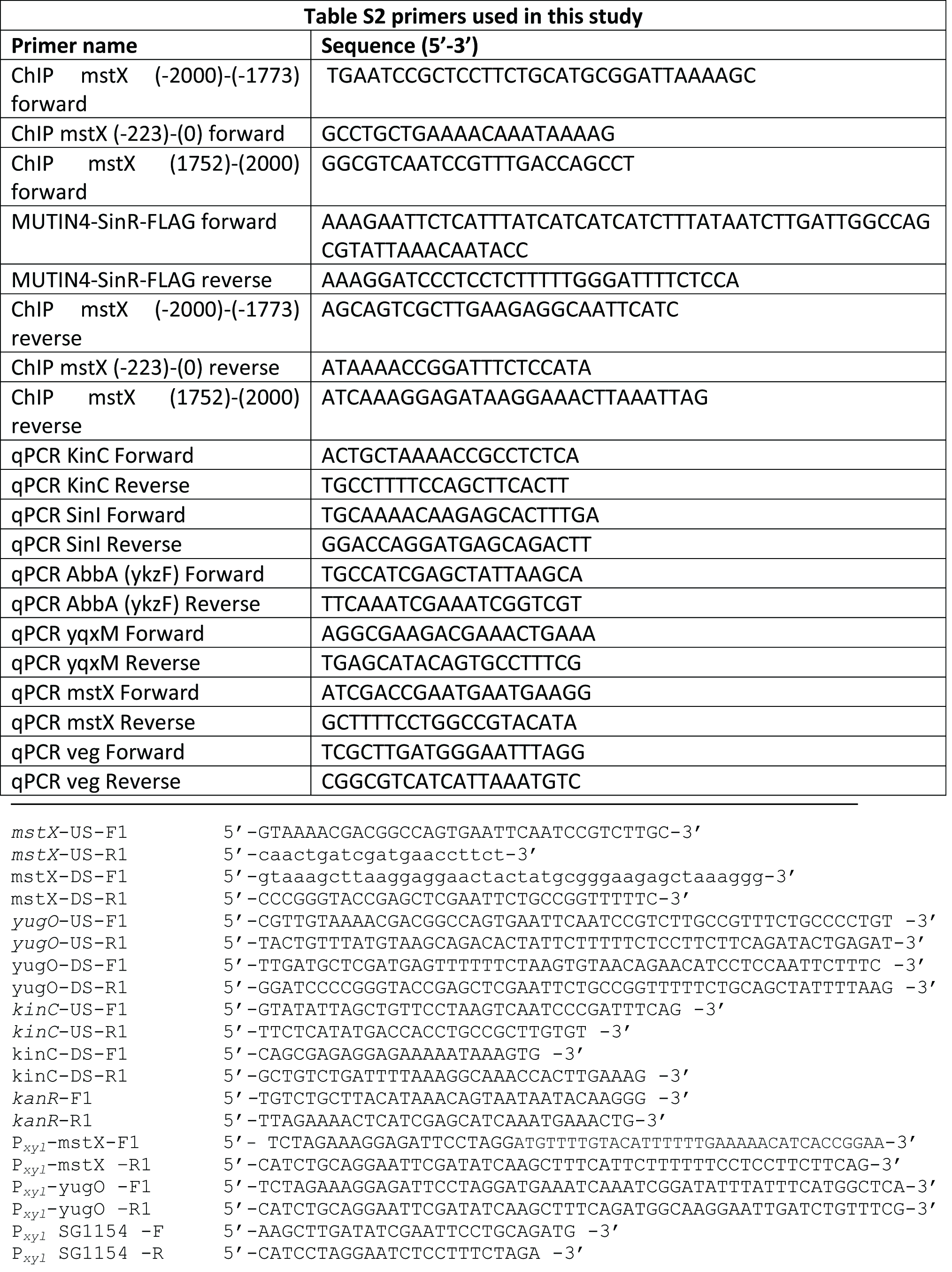

Supplement: Table S2 — Primers used in this study. (TIF) [file pone.0060993.s004.tif]
