# Supplementary material for: MstX and a Putative Potassium Channel Facilitate Biofilm Formation in Bacillus subtilis
Source: PLoS One. 2013 May 30;8(5):e60993. doi: 10.1371/journal.pone.0060993 (PMC3667857; doi:10.1371/journal.pone.0060993)
Supplement: Text S1 — Materials and methods. (DOCX) [file pone.0060993.s005.docx]

**Materials and Methods**

**Plasmids and strains**

*B. subtilis* strains were derivatives of wild-type strain PY79 and NCIB3610 strains and are listed in Table S1. Unless otherwise noted, mutations were constructed by long flanking homology PCR [1] and introduced into the domesticated strain PY79 by transformation. Plasmid constructions were performed in *E. coli* DH5α using standard methods. Transduction was used to move mutations from PY79 to NCIB3610 [2].

The *mstX* deletion mutants were constructed by using LFR-PCR to replace the *mstX* coding region with the *loxP-kan-loxP* cassette [3]. A ribosomal binding site was introduced upstream of the *yugO* translational initiation site as described (Becker et al. [3]) as a means of preventing the translational disruption of *yugO* in the *mstX* knockout strain. The resulting plasmid was transformed into PY79, where it integrated into *mstX* to create MEL110. For MEL64, MEL110 was treated with Cre recombinase *in vivo* [3] to produce ∆*mstX* and remove the *kanR* cassette.

For MEL111, long-flanking homology PCR was used to introduce a *kan* cassette into *yugO*. Colonies were screened to confirm double-crossover recombination and disruption of *yugO*.

For the IPTG-inducible *mstX* construct MEL66, full-length wild type *mstX* was cloned into pMUTIN4 [4] and integrated ectopically at the *lacA* locus to create *P_spac_-mstX* *erm.* For MEL67, full-length *mstX* containing a point mutation (M75A) was cloned into ECE138 (BGSC) and integrated at the *lacA* locus to create *P_spac_-mstX* M75A *erm*. The point mutation was introduced via a modified version of the Quikchange method [5].

For MEL102, the *sinR* gene encoding a C-terminal FLAG fusion to SinR was cloned into ECE139 pMUTIN4 [4]. The resulting construct was then transformed into PY79 selecting for erythromycin resistance, introducing a *sinR-FLAG* gene through single-crossover recombination.

For MEL73, long-flanking homology PCR was used to introduce a *neo* cassette into *sinR* [1]. Colonies were screened by PCR to confirm double-crossover recombination and disruption of *sinR*. For MEL212, long-flanking homology PCR was used to introduce a *spc* cassette into *kinC* by double recombination.

For MEL112, long-flanking homology PCR was used to introduce a *spc* cassette into *kinC*. Colonies were screened to confirm double-crossover recombination and disruption of *kinC*.

For MEL217, long-flanking homology PCR was used to introduce a *cam* cassette into *kinC*. Colonies were screened to confirm double-crossover recombination and disruption of *kinC*.

For MEL218, long-flanking homology PCR was used to introduce a *spc* cassette into *sinR*. Colonies were screened to confirm double-crossover recombination and disruption of *sinR*.

For MEL239, chromosomal DNA from MEL64 was introduced into NCBI 3610 via phage transduction using standard protocols [2].

For MEL240, chromosomal DNA from MEL110 was introduced into NCBI 3610 via phage transduction using standard protocols [2].For MEL420, the wild type *mstX* gene with a stop codon was cloned into pSG1154 (BGSC) [6] and integrated ectopically at the *amyE* locus to create *amyE::P_xyl_-mstX::spc* in the MEL64 background strain (PY79 ∆*mstX::loxP*). The mutant was confirmed for no loss of *kan*.

For MEL421 *P_xyl-_mstX ΔyugO*, chromosomal DNA was prepared from MEL420 and introduced into MEL239. Mutants were selected based for *spc*. For MEL422, chromosomal DNA was prepared from MEL420 and introduced into MEL240. Mutants were selected based for *spc*.

For MEL423, chromosomal DNA from MEL218 was introduced into NCBI 3610 via phage transduction using standard protocols [2].

For MEL424, chromosomal DNA from MEL423 was introduced into MEL239 via phage transduction using standard protocols [2]. Mutants were selected based upon *spc*.

For MEL425, chromosomal DNA from MEL423 was introduced into MEL240 via phage transduction using standard protocols [2]. Mutants were selected based upon *spc*.

For MEL425, chromosomal DNA from MEL217 was introduced into NCBI 3610 via phage transduction using standard protocols [2].

For MEL426, chromosomal DNA from MEL112 was introduced into NCBI 3610 via phage transduction using standard protocols [2].

For MEL427, chromosomal DNA from MEL217 was introduced into NCBI 3610 via phage transduction using standard protocols [2].

For MEL428, chromosomal DNA from MEL423 was introduced into MEL427 via phage transduction using standard protocols [2]. Mutants were selected based upon *spc.*

For MEL429, chromosomal DNA from MEL217 was introduced into MEL422 via phage transduction using standard protocols [2]. Mutants were selected based upon *cam*.

For MEL430, chromosomal DNA from MEL432 was introduced into MEL239 via phage transduction using standard protocols [2]. Mutants were selected based upon *spc*.

For MEL431, chromosomal DNA from MEL432 was introduced into MEL240 via phage transduction using standard protocols [2]. Mutants were selected based upon spc resistance.

For MEL432, LFH-PCR and Gibson cloning was used to introduce *yugO* into pSG1154 under control of a xylose-inducible promoter [6,7]. The resulting plasmid construct was introduced into MEL63 by ectopic integration at *amyE.*


**Supplementary references**

[1] Wach, A. 1996. PCR-synthesis of marker cassettes with long flanking homology regions for gene disruptions in S. cerevisiae. *Yeast* **12**: 259-265.

[2] Branda, S.S., Gonzalez-Pastor, J.E., Ben-Yehuda, S., Losick, R., and Kolter, R. 2001. Fruiting body formation by *Bacillus subtilis*. *PNAS* **98**: 11621-11626.

[3] Becker, E., Herrera, N.C., Gunderson, F.Q., Derman, A.I., Dance, A.L., Sims, J., Larsen, R.A., and Pogliano, J. 2006. DNA segregation by the bacterial actin AlfA during Bacillus subtilis growth and development. *EMBO J* **25**: 5919-5931.

[4] Vagner, V., E. Dervyn, and S.D. Ehrlich. 1998. A vector for systematic gene inactivation in *Bacillus subtilis*. *Microbiology* **144**: 3097-3104.

[5] Sawano, A. and Miyawaki, A. (2000) Directed evolution of green fluorescent protein by a new versatile PCR strategy for site-directed and semi-random mutagenesis. *Nucleic Acids Res* **28**: 78.

[6] Lewis, P.J. and A.L. Marston. 1999. GFP vectors for controlled expression and dual labeling of protein fusions in *Bacillus subtilis*. *Gene* **227**: 101-109.

[7] Gibson, D.G., Young, L., Chuang, R.Y., Venter, J.C., Hutchison, C.A. 3rd, and Smith, H.O. 2009. Enzymatic assembly of DNA molecules up to several hundred kilobases. *Nature Methods* **6**: 343-345.
